# Electrophysiological Evidence of Event-Related Potential Changes Induced by 12 h Abstinence in Young Smokers Based on the Flanker Study

**DOI:** 10.3389/fpsyt.2020.00424

**Published:** 2020-05-22

**Authors:** Yongting Cui, Fang Dong, Xiaojian Li, Dongdong Xie, Yongxin Cheng, Shiyu Tian, Ting Xue, Yangding Li, Ming Zhang, Yan Ren, Kai Yuan, Dahua Yu

**Affiliations:** ^1^Inner Mongolia Key Laboratory of Pattern Recognition and Intelligent Image Processing, School of Information Engineering, Inner Mongolia University of Science and Technology, Baotou, China; ^2^College of Information Science and Engineering, Hunan Normal University, Changsha, China; ^3^Life Sciences Research Center, School of Life Science and Technology, Xidian University, Xi’an, China

**Keywords:** error processing, adolescent smokers, abstinence, event-related potential (ERP), performance monitoring

## Abstract

The cognitive control processes may be disrupted by abstinence in smokers, which may be helpful in the development and maintenance of addictive behavior. The purpose of this study was to measure the performance of cognitive task after 12 h of smoking abstinence by using event-related potentials (ERPs), including the error-related negativity (ERN) and the error positivity (Pe). In Eriksen flanker task, electroencephalography (EEG) signals of 24 smokers were recorded in two conditions: satiety and 12 h abstinence. In the behavioral data, both conditions exhibited more errors and more time on the incongruent trials than congruence. Meantime, the Minnesota Nicotine Withdrawal Scale (MNWS) score was increased during abstinence. Smokers showed reduced ERN and Pe after 12 h of abstinence, compared with satiety condition. The results indicate that the diminished error processing in young smokers after 12 h of abstinence. It may be related to increased withdrawal symptoms. In conclusion, the disrupted neurophysiological indexes in the general behavior monitoring system may be caused by abstinence. The results of this study may provide us with new ideas about the effects of short-term abstinence on brain cognitive neuroscience and be helpful for the solution of relapse.

## Introduction

According to statistics from the Global Youth Tobacco Survey (GYTS) in 2014, the rate of attempted smoking among adolescents in China was 19.9% (30.1% for boys and 8.7% for girls, 82.3% of them tried smoking for the first time before the age of 13), the current smoking rate (the smoking behavior in the past month) is 6.9% (11.2% for boys and 2.2% for girls) (1). In addition, at least 20% of young smokers with high nicotine dependence will develop into ordinary smokers (2). Previous studies have shown that the brain structure and nervous system development of adolescent smokers may be affected by nicotine (3–7). But most attempts to quit ended in relapse, even though they realized the adverse consequences of smoking and expressed their strong desire to quit (8).

Abstinence from smoking disrupts cognitive performance that may help maintain smoking behavior (9, 10). Previous studies reported that a general performance monitoring system involving a range of cognitive functions after acute abstinence from smoking may be a neurophysiological indicator (11). Besides, our group research showed that the interaction of neurological mechanisms can be significantly changed, which affects the balance between cognitive control and reward (8, 12–15). Studies have shown that most smokers are prone to relapse within 24 h of withdrawal (16) and have a series of withdrawal symptoms (17).

The core function of cognitive control is to monitor and regulate our behavior. In this study, we investigated the error monitoring, as an aspect of cognitive control, which plays an important role in behavioral regulation and cognitive control (18–20). Using two event-related potential (ERP) components associated with error processing: the error-related negativity (ERN or Ne) and the error positivity (Pe), which appears after an error and seems to be sensitive (21). Researches particularly relevant to this study have been shown the reduction of ERN and Pe, indicating that initial error processing and motivational significance of smokers are affected after errors. Few ERP studies have investigated performance monitoring in substance dependence (22–25). Meantime, there are few studies to investigate the error monitoring of young smokers under the abstinence condition. Nor can we identify any studies investigating the link between error processing and smoking abstinence.

Previous neurophysiological studies conducted that lower ERN and Pe amplitudes during abstinence in adults (11), suggested that the neurophysiological indicators of performance monitoring are affected in the process of acute abstinence. Based on these results, we expect to observe the decrease of ERN or Pe during abstinence condition by using two ERP components related to error monitoring: ERN and Pe. In conclusion, the main purpose of this study is to test the prediction of whether the general performance monitoring system would be disrupted after overnight abstinence from smoking.

## Materials And Methods

### Ethics Statement

The study was approved by the Medical Ethical Committee of the First Affiliated Hospital of Baotou Medical College, Inner Mongolia University of Science and Technology, and was in agreement with the Declaration of Helsinki. All procedures were carried out with the adequate understanding and written informed consent of the subjects and their legal guardian after understanding the purpose of the study.

### Participants

Participants were 24 young male smokers recruited from Inner Mongolia University of Science and Technology by using Internet and print media advertisements in the present study. All participants are right-handed. Mean age of the smokers was 21.04 ± 1.27 (mean ± SD). The criteria for inclusion of all young smokers are as follows: 1) correspond with DSM-V criteria for current nicotine dependence; 2) consumed 10 or more cigarettes per day in the last 6 months; 3) ≥3 points on the Fagerstrom Test for Nicotine Dependence (FTND) (26); 4) no period of smoking abstinence longer than 3 months; 5) expired air carbon monoxide (CO)>6 ppm (ppm) (by Smokelyzer, Bedfont Scientific, Kent, UK); 6) there are currently no physical, neurological, or psychiatric or extracranial lesions assessed by alcohol or substance abuse. **Table 1** presents the demographic and smoking characteristics of the sample. Several standards and widely used self-measures were completed prior to or after the lab session. In this study, 10 items of smoking impulse questionnaire (QSU) were used to measure the craving level of subjects (27). Current nicotine withdrawal was assessed before and after the 15 min testing session using a revised Minnesota Nicotine Withdrawal Scale [MNWS; (28)]. Breath carbon monoxide (CO) was measured before the testing session.

**Table 1:** Demographic and smoking characteristics of young smokers.

Clinical details	Young smokers (n = 24)
Age	21.04 ± 1.27
Age range (years)	19-24
Education (years)	14.13 ± 0.61
Age of smoking initiation	15.33 ± 2.78
Smoking duration (years)	4.21 ± 2.21
Pack-years	2.95 ± 2.73
Cigarettes per day (CPD)	14.25 ± 4.59
FTND total score	4.75 ± 1.48

Values are expressed as means ± standard deviations.

FTND, Fagerström Test for Nicotine Dependence.

Pack-years: smoking years × daily consumption/20.

### Procedure

Participants completed the demographic questionnaire before the experiment began. We designed two identical EEG sessions in 1–3 weeks (1) smoking as usual (satiety) and (2) ≥ 12-h abstinence (abstinence). The time interval between withdrawal and EEG data collection is ~12 h. During satisfied condition, smokers can smoke their first cigarette of the day (of their preferred brand) within 30 min before the experiment. All participants were asked to ensure adequate sleep before the experiment. The specific CO measurement standard is to measure the CO level concentration of the participants within 10 min before the start of the experiment. In particular, under the state of abstinence, the participants were required to tell the time of the last cigarette. Abstinence was verified by a carbon monoxide level of less than 6 ppm (29). Expired air carbon monoxide (CO) readings were obtained using the Smokerlyzer system (Bedfont Scientific Ltd., Rochester, UK) in two sessions before the beginning of the experiment. Meanwhile, participants completed the MNWS and Brief-QSU scales. Participants were placed in a comfortable environment free from outside interference. Participants then sat in comfortable chairs and electrodes were attached. And participants are told that errors are inevitable, but they need to complete the task as quickly and correctly as possible and pay attention during the task. Then the participants began to complete the Flanker task. The study used E-Prime 2.0 software to present stimuli and collect behavioral data. Participants were remunerated in the study.

### Task Paradigm

**Figure 1:**
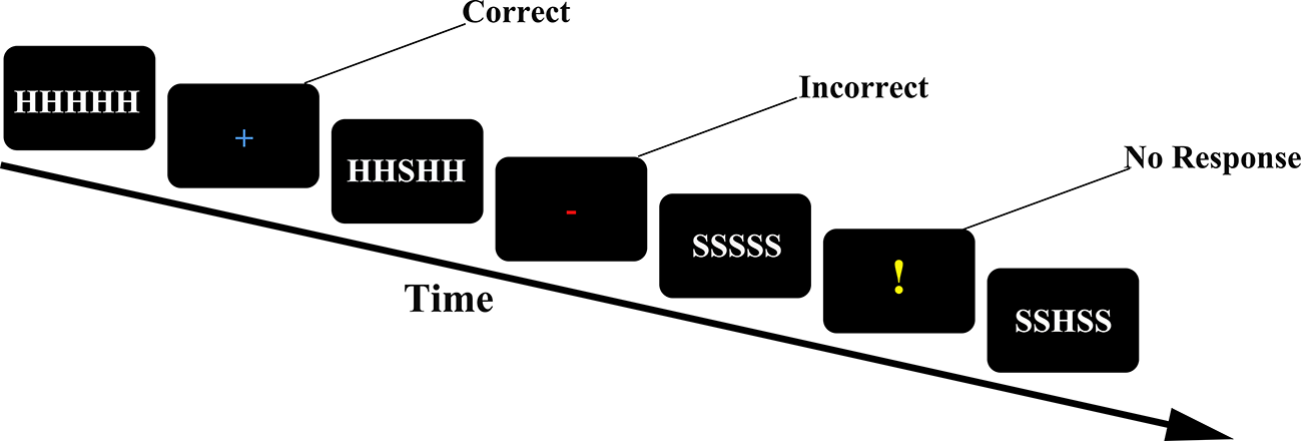
Trial structure of the flanker task. Feedback was informative (“+”for correct; red for incorrect; “!” for missing or not responding in time).

Task paradigm the Eriksen flanker task (30) takes the most typical subtitle stimulus paradigm as an example. The experimenter provides participants with a series of five-letter strings. Four different strings (SSSSS SSHSS, HHHHH HHSHH) appear in the center of the computer screen. If the middle letter is H, the participant presses a button with the right index finger. If the middle letter is S, the participant presses a button with the left index finger. When the participant responds correctly, a “+” will be feedback in the center of the screen; when the participant responds incorrectly, a red rectangle will be received; when the participant does not respond, a “!” will be feedback in the center of the screen. The entire task consists of five groups, each group will present 80 strings, and the number of occurrences of the congruent group and the incongruent group is the same, 40 times (n = 40). Since the string in the center of the screen is presented for a very short time, participants are required to respond quickly to the stimulus (**Figure 1**).

### Electroencephalography Recording and Data Analysis

All EEG data were collected in a quiet, dimly lit, and good sound-attenuated EEG laboratory. Recording EEG data with BrainAmp MR plus amplifier (Brain Products GmbH. Munich. Germany) from 64 scalp sites (positioned following the 10–20 International System) with Ag/AgCl electrodes mounted in a cap (EasyCap, GmbH). Stimuli were presented on a computer using E-Prime 2.0 software and about 45 cm away from the participants. Two active Ag/AgCl electrodes were placed in the outer canthus of right eye and above the left eye to record the vertical electrooculogram (EOG). All signals were digitalized with a sample rate of 1,000 Hz with a frequency band from 0.15 to 35 Hz [infinite impulse response (IIR) filter 24/dB/octave roll of], and electrode impedances were reduced to less than 10 kΩ. BrainVision Analyzer 2 (Brain Products GmbH. Munich. Germany) software is used to analyze all offline data. TP9 and TP10 were employed as re-referenced and the FCz electrode was a reference electrode. The EEG signals were segmented in 1,000 ms epochs (200 ms before and 800 ms after response) and artifact rejection was done for all epochs. The rejection criteria for all epochs were: 1) maximal allowed voltage skip (gradient) is 50 μV for each ample point; 2) minimum allowed activity 0.5 μV in a 100 ms interval and maximal allowed value difference 200 μV in a 200 ms interval. The independent component analysis (ICA) was applied to remove eye movements and eye blinks. The mean 200 ms pre-response period was served as baseline. After baseline correction, average ERP waves were calculated for artifact-free trials at each scalp site in the two response (correct and incorrect). The ERN and Pe amplitudes were defined as the mean value at the signal subject level (ERN, 50–150 ms; Pe, 200–400 ms) (22, 31, 32) after onset of the response. For both the ERN and Pe, we studied the midline electrodes Fz, Cz, and Pz.

### Statistics

All data are analyzed and processed by SPSS 20.0 software (SPSS Statistics, IBM, Armonk, NY). Two-sample paired t-test was employed to compare demographic data [pack-years, FTND, cigarettes per day (CPD)], questionnaires score, behavior performance (the number of errors, reaction time), and ERP data (amplitude and latency of the ERN and Pe) differences between the satiety and abstinence condition. Eventually, calculate the Pearson correlation coefficients between them.

## Results

### Questionnaires and Breath Carbon Monoxide-Concentration Analysis

**Figure 2:**
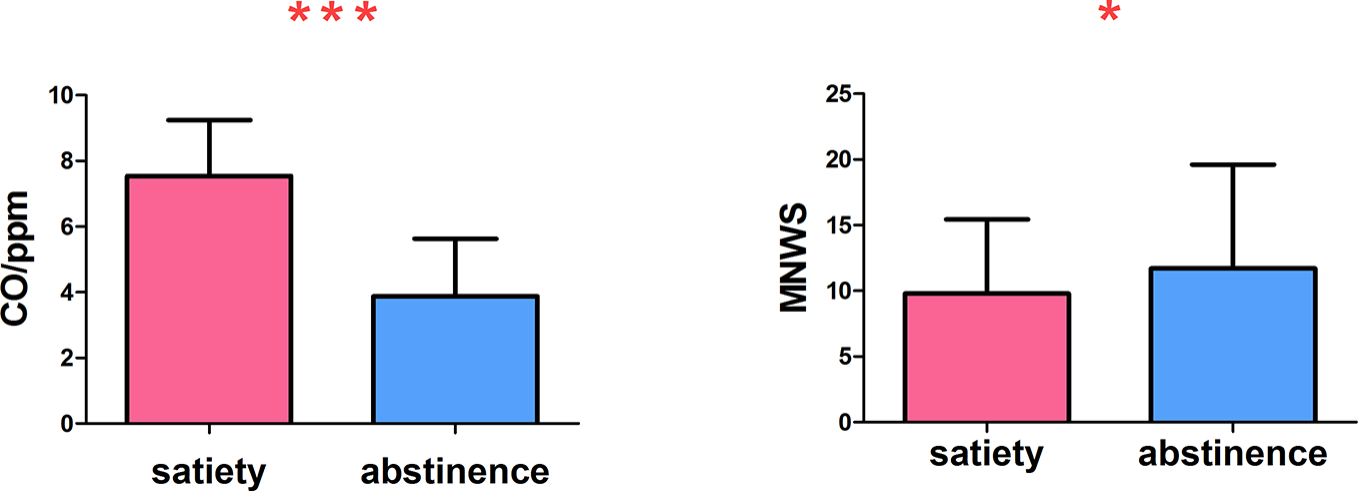
The carbon monoxide in expired air was significantly reduced and the withdrawal symptoms increased *p < 0.05, ***p < 0.001. * and *** Represents that there were significant differences in CO level and withdrawal symptoms between satiety and abstinence in smokers.

During abstinence, carbon monoxide in the expired air was significantly lower (t = −9.326, p <.000). On the MNWS assessment of withdrawal symptoms, the score was increased during abstinence (t = 2.172, p =.040) (**Figure 2**).

### Behavioral Data

**Figure 3:**
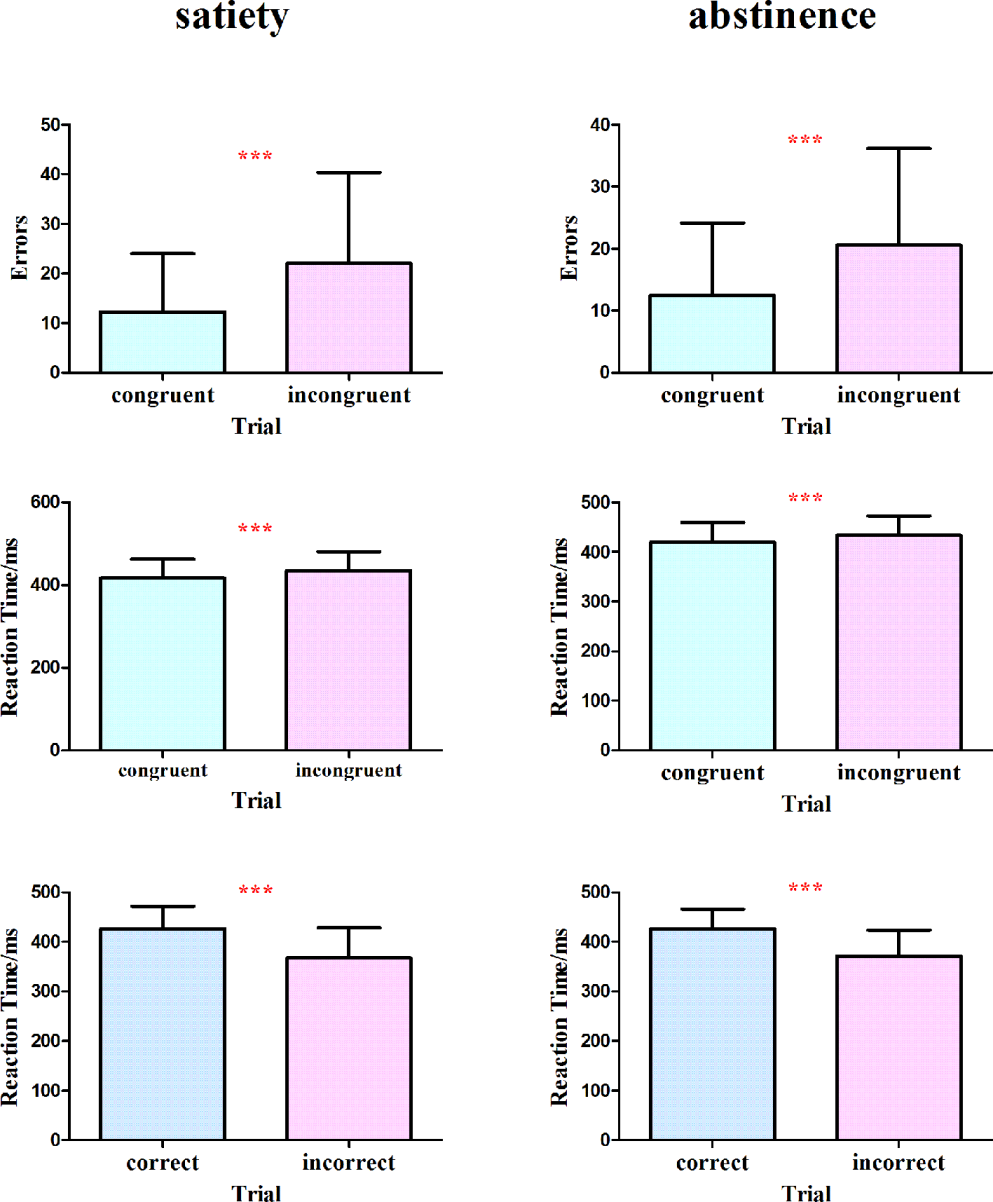
No significant difference was found in the total number of errors between the smoking satiety condition and the 12-h abstinence smoking condition (t = −.182, p = .858). More errors were found on the incongruent trials than on the congruent (t = −7.560, p < .000) and responses were markedly faster on congruent trials (t = −9.842, p < .000) in both two conditions. Moreover, it took more time to respond on the correct trials as compared to incorrect trials (t = 10.993, p < .000). ***p < 0.001. *** Represents that there are significant differences in the number of errors and reaction time between different trial conditions.

**Table 2** shows the number of errors and reaction time both two conditions made on the flanker task. The total number of errors was similar between the smoking satiety condition and the 12-h abstinence smoking condition (t = −.182, p =.858). However, there are more errors on the incongruent trials than on the congruent trials (t = −7.560, p <.000) in both conditions. Responses were markedly faster on congruent trials (t = −9.842, p <.000). Moreover, it spent more time to respond on the correct trials as compared to incorrect trials (t = 10.993, p <.000) (**Figure 3**).

**Table 2:** Means and standard deviations for task performance variables.

	Satiety (n=24)	12-h abstinence (n=24)	p-Value
Number of errors of all response	40.17 ± 23.31	40.96 ± 27.78	0.858
Number of errors on congruent trails	12.25 ± 11.78	12.46 ± 11.72	0.851
Number of errors on incongruent trails	22.04 ± 18.35	20.58 ± 15.63	0.527
Correct	425.87 ± 46.42	426.67 ± 39.31	0.869
Incorrect	367.87 ± 60.55	371.34 ± 52.53	0.734
Congruent	416.13 ± 46.77	419.64 ± 40.03	0.486
Incongruent	434.66 ± 45.95	433.48 ± 39.28	0.809

Values are expressed as means ± standard deviations.

### Electroencephalography Data

#### Error-Related Negativity

**Figure 4:**
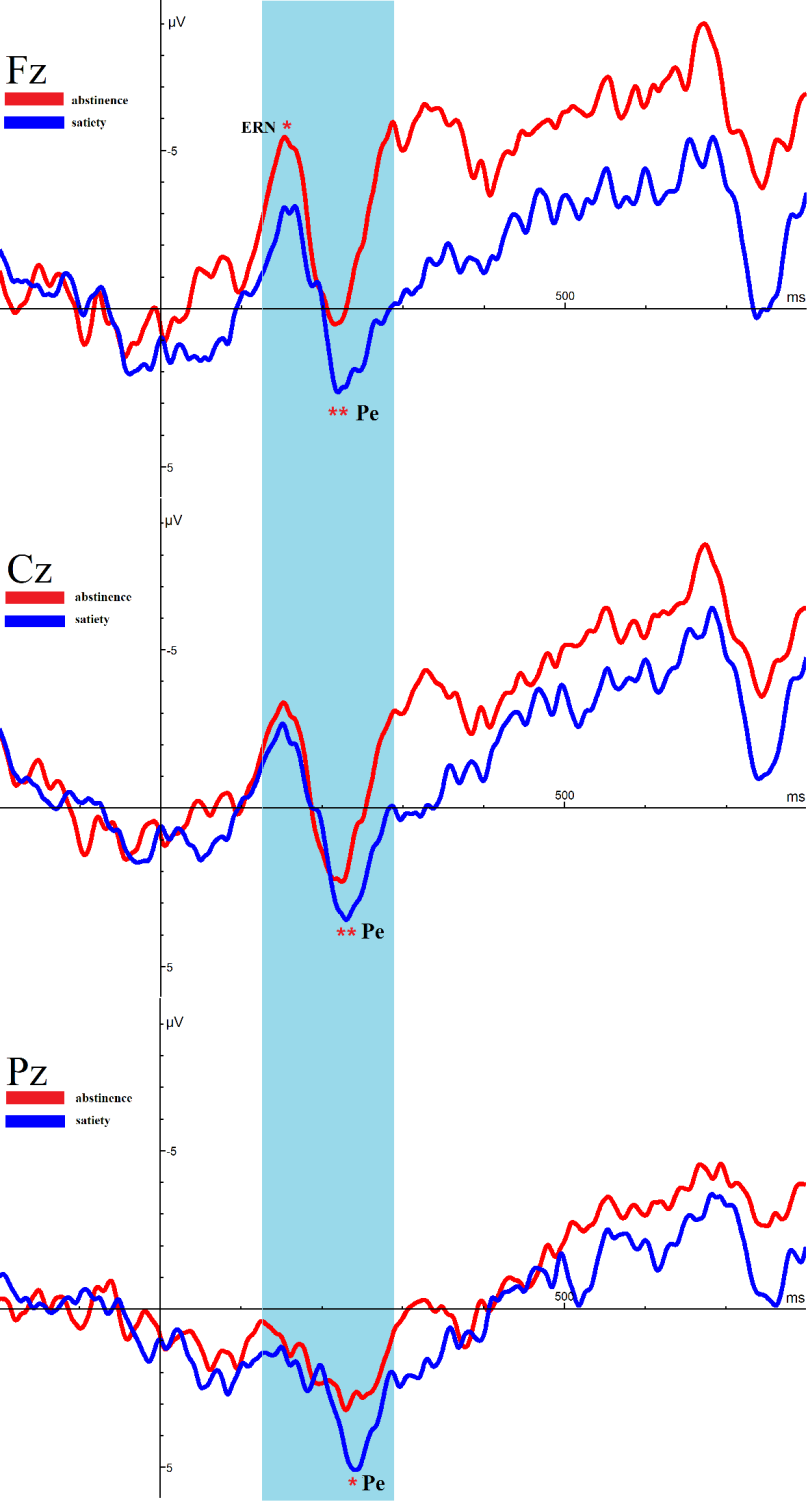
Average event-related potential (ERP) activity during error trials of the Eriksen Flanker Task for satiety and abstinence. *p < 0.05, **p < 0.01. * and ** Represents an area where there are significant differences in ERP between satiety and abstinence in young smokers.

**Figure 4** shows the ERN and Pe amplitude of both conditions on the incorrect trials at the midline electrodes Fz, Cz, and Pz. After 12 h of abstinence, the ERN was significantly decreased at Fz (t = −2.268, p =.033), but no differ at Cz (t = −1.668, p =.109) and Pz (t = −.976, p =.339) on the incorrect trials.

#### Error Positivity

Remarkably, the amplitude of Pe in 12-h smoking abstinence condition was reduced compared with smoking satiety conditions in young smokers (Fz: t = −3.084, p =.005, Cz: t = −2.974, p =.007, Pz: t = −2.562, p =.016).

### Correlations Between Smoking Variables, Behavioral Data, and Event-Related Potentials

There was no significant correlation between any of the smoking clinical data, behavioral data, and ERPs.

## Discussion

The smoking rate of adolescents in China is increasing continuously, and the age of smoking tends to be younger, which may promote nicotine dependence in later life. In the present study, the error-related negativity (ERN) and error positivity (Pe) were used as indicators to test the effect of overnight smoking abstinence on performance monitoring. Compared with smoking as usual, Pe amplitude decreased significantly during abstinence condition, which was consistent with our prediction. More specifically, this study showed the ERN amplitude was significantly decreased after 12 h of abstinence. Our findings may reveal electrophysiological evidence for the reduction error processing in the flanker task during abstinence condition. In addition, the level of self-reported withdrawal symptoms was higher after 12 h abstinence, which was not associated with the reduction in the ERN and Pe brain potentials. On the behavioral level, more errors were shown in the two conditions of incongruent trials, and more reaction time was spent in the incorrect trials. Participants abstinence for at least 12 h and the carbon monoxide in expired air was significantly reduced.

The ERN appears immediately after committing errors that peaks at approximately 50–150 ms after error commission. Although researchers lack of consensus on the specific functional significance of ERN, some believe that ERN may reflect the early, automatic, and unconscious processing of errors (33). To test the effect of abstinence on one aspect of cognitive performance, ERN may be a useful indicator (34). In the present study, we found that the ERN amplitude was significantly decreased at Fz after making an error. Several studies have shown that there are neural systems in the medial frontal cortex (MFC) for monitoring errors, including the rostral cingulate motor area, the supplementary eye field, and the dorsal anterior cingulate cortex (dACC) (35). A study documenting both EEG and functional MRI (fMRI) more strongly shows that there is a strong link between ERN and the activation of error-related ACC (36). Previous fMRI studies have shown that ERN and Pe are produced by the anterior cingulate cortex, and plays an important role in motivation and regulation problems. Therefore, the decreased of ERN at Fz electrode in the state of abstinence reflects the decrease in error processing. Consistent with this interpretation, Luijten *et al*. found young smokers with modestly deprived healthy had a reduced ERN compared to a non-smoker control group. Several other studies were also in accordance with our findings, such as cocaine (24) and juvenile violent offenders (37), suggesting that a deficit in performance monitoring. All of the above reveals that reduced ERN is associated with error processing. In a previous study on smoking, Franken et al. (22) showed that the amplitude of ERN did not decrease, indicating that the initial error processing seems not to be disrupted. However, Mayra L. Padilla et al. (38) showed that larger ERN amplitudes improved performance monitoring enabling abstinent alcoholics to overcome response conflicts. The differences found in these studies may be due to differences in dependent substances (nicotine *vs.* cocaine, resp.) or abstinence states (satiety *vs.* abstinent) (22).

Immediately after ERN, a positive ERP component (error positivity, Pe) appeared, which peaks at approximately 200–400 ms after the error (39). The Pe is considered to represent a conscious, more elaborate post-processing of an error (39). Although the role of cognition in nicotine dependence has aroused great interest (40), and in the cognitive neuroscience of the Pe (37, 41–43), it seems to be the first study to show that Pe is sensitive to acute abstinence from smoking. Previous ERP studies have shown that the Pe possibly reflects the error awareness (44), motivational significance (45), or emotional assessment of an error (46). Of particular relevance to the present study, some studies found smokers are affected by the initial motivational significance attributed to an error during smoking cue exposure (47). As expected, we found that the Pe amplitude of smokers decreased after 12 h abstinence. The current findings converge with a previous study that the Franken et al. (22) found that a decrease in Pe amplitude suggests that smoking is associated with a reduction in error processing. Meantime, they found that the Pe amplitude was reduced in cocaine dependent subjects as compared to healthy controls, suggesting that cocaine addiction was related to error processing (23). In addition, Luijten M et al. (47) found the same results and suggests that this may contribute to the development and maintenance of addictive behaviors. Some other studies also have confirmed that the change of Pe reflects the abnormality of performance monitoring (32, 48). These findings again demonstrate a reduced error processing after 12 h of abstinence, and may contribute to the maintenance of addictive behavior. Unexpectedly, we did not find significant relationship between behavioral data and the Pe. This may be due to the short withdrawal time. Furthermore, we observed the withdrawal (MNWS) was increased during abstinence. Smokers commonly report that difficulty concentrating during smoking abstinence (28), and standard assessments of withdrawal include cognitive items (e.g., Minnesota Nicotine Withdrawal Scale). The current results seem to be associated with smoking abstinence. Previous studies found that abstinence led to reduced heart rate, feelings of depression, irritability, poor task performance, stress, poor concentration, restlessness, and smoking impulses (49). This may be provided the idea for explaining the reduction of performance monitoring under abstinence condition.

In sum, results of the present study showed the diminished error processing in young smokers after 12 h of abstinence at the physiological level. At the same time, reduced error processing might be the result of abstinence. It reveals that abstinence may disrupt the neurophysiological indicators of performance monitoring. These results supplement the extant literature. Importantly, this may be the first study to assess and demonstrate the effect of neurophysiological indicators of performance monitoring in young smokers after short-time abstinence. The results of this study are helpful to understand the cognitive and motivational factors of relapse behavior after abstinence from smoking.

### Limitations

First, the current study is the relatively small size of the samples, and the participants were all men in this study. Second, at present, smokers are young smokers with a low degree of smoking dependence, and they are relatively in the early stage. The effect of abstinence may be more significant in more serious smokers. In the future, more subjects should be added in the research, while smokers with severe nicotine dependence are better.

## Data Availability Statement

The datasets generated for this study will not be made publicly available because there are multiple collaborating units, and research and experiments have not been completed, the data cannot be made public for other researchers to share. Requests to access the datasets should be directed to the corresponding authors.

## Ethics Statement

The studies involving human participants were reviewed and approved by the Medical Ethical Committee of the First Affiliated Hospital of Baotou Medical College, Inner Mongolia University of Science and Technology. The patients/participants provided their written informed consent to participate in this study.

## Author Contributions

DY, KY, and YL conceived and designed the experiments. XL, DX, YCh, TX, ST, and MZ performed the experiments. YCu, FD, XL, and YCh analyzed the data. YCu and FD wrote the manuscript. TX, YR, DY, and KY provided the critical revision of the manuscript. All authors critically reviewed the content and approved the final version for publication.

## Funding

This work is supported by the National Natural Science Foundation of China under Grant Nos. 81871430, 81871426, 61771266, 81701780, 31800926, 81401478, 81470816, 81471737, the Fundamental Research Funds for the Central Universities under the Grant No. JB151204, the program for Young adult Talents of Science and Technology in Universities of Inner Mongolia Autonomous Region NJYT-17-B11, the Natural Science Foundation of Inner Mongolia under Grant No. 2019JQ07, 2017MS(LH)0814, 2018LH08079. The program of Chunhui in Ministry of Education of China 2018-45, The Natural Science Basic Research Plan in Shaan-xi Province of China under Grant No. 2018JM7075. US National Institutes of Health, Intramural Research program Y1AA3009.

## Conflict of Interest

The authors declare that the research was conducted in the absence of any commercial or financial relationships that could be construed as a potential conflict of interest.

## References

[1] Warren CW Riley L Asma S Eriksen MP Green L Blanton C Tobacco use by youth: a surveillance report from the Global Youth Tobacco Survey project. Bull World Health Organ (2000) 78(7):868–76. 10.1590/S0042-96862000000700003 PMC2560802 10994259

[2] Lantz PM Smoking on the rise among young adults: implications for research and policy. Tobacco Control (2003) 12(suppl 1):i60–70. 10.1136/tc.12.suppl_1.i60 PMC1766087 12773786

[3] Su S Yu D Cheng J Chen Y Zhang X Guan Y Decreased Global Network Efficiency in Young Male Smoker: An EEG Study during the Resting State. Front Psychol (2017) 8:1605. 10.3389/fpsyg.2017.01605 28951727 PMC5599785

[4] Yuan K Yu D Bi Y Li Y Guan Y Liu J The implication of frontostriatal circuits in young smokers: A resting-state study. Hum Brain Mapp (2016) 37(6):2013–26. 10.1002/hbm.23153 PMC6867544 26918784

[5] Yuan K Yu D Zhao M Li M Wang R Li Y Abnormal frontostriatal tracts in young male tobacco smokers. NeuroImage (2018) 183:346–55. 10.1016/j.neuroimage.2018.08.046 30130644

[6] Li Y Yuan K Cai C Feng D Yin J Bi Y Reduced frontal cortical thickness and increased caudate volume within fronto-striatal circuits in young adult smokers. Drug Alcohol Depend (2015) 151(2015):211–9. 10.1016/j.drugalcdep.2015.03.023 25865908

[7] Yu D Yuan K Cheng J Guan Y Li Y Bi Y Reduced thalamus volume may reflect nicotine severity in young male smokers. Nicotine Tobacco Res (2017) 20(4):434–9. 10.1093/ntr/ntx146 28651369

[8] Bi Y Zhang Y Li Y Yu D Yuan K Tian J 12h abstinence-induced right anterior insula network pattern changes in young smokers. Drug Alcohol Depend (2017) 176:162–8. 10.1016/j.drugalcdep.2017.02.019 28544994

[9] Zhao H Turel O Brevers D Bechara A He Q Smoking cues impair monitoring but not stopping during response inhibition in abstinent male smokers. Behav Brain Res (2020) 386, 112605. 10.1016/j.bbr.2020.112605 32179061

[10] Liu C Dong F Li Y Ren Y Xie D Wang X 12h abstinence-induced ERP changes in young smokers: electrophysiological evidence from a Go/NoGo study. Front Psychol (2019) 10:1814. 10.3389/fpsyg.2019.01814 31474901 PMC6703154

[11] Schlienz NJ Hawk LW Rosch KS The effects of acute abstinence from smoking and performance-based rewards on performance monitoring. Psychopharmacology (2013) 229(4):701–11. 10.1007/s00213-013-3131-8 PMC3784636 23681159

[12] Li Y Yuan K Bi Y Guan Y Cheng J Zhang Y Neural correlates of 12-h abstinence-induced craving in young adult smokers: a resting-state study. Brain Imaging Behav (2016) 11(3):677–84. 10.1007/s11682-016-9544-3 26995747

[13] Kai Y Meng Z Yu D Manza P Volkow ND Wang GJ Striato-cortical tracts predict 12-h abstinence-induced lapse in smokers. Neuropsychopharmacology (2018) 43(12):2452–8. 10.1038/s41386-018-0182-x PMC6180048 30131564

[14] Zhao S Li Y Li M Wang R Bi Y Zhang Y 12-h abstinence-induced functional connectivity density changes and craving in young smokers: a resting-state study. Brain Imaging Behav (2018) 13(4):953–62. 10.1007/s11682-018-9911-3 29926324

[15] Cheng J Guan Y Zhang Y Bi Y Bu L Li Y Electrophysiological mechanisms of biased response to smoking-related cues in young smokers. Neurosci Lett (2016) 629:85–91. 10.1016/j.neulet.2016.06.062 27373532

[16] Zelle SL Gates KM Fiez JA Sayette MA Wilson SJ The first day is always the hardest: Functional connectivity during cue exposure and the ability to resist smoking in the initial hours of a quit attempt. Neuroimage (2016) 151:24–32. 10.1016/j.neuroimage.2016.03.015 26975550 PMC5018416

[17] Allen SS Bade T Hatsukami D Center B Craving, withdrawal, and smoking urges on days immediately prior to smoking relapse. Nicotine Tobacco Res (2008) 10(1):35–45. 10.1080/14622200701705076 18188743

[18] Ridderinkhof KR Wildenberg WPMvd Segalowitz SJ Carter CS Neurocognitive mechanisms of cognitive control: the role of prefrontal cortex in action selection, response inhibition, performance monitoring, and reward-based learning. Brain Cogn (2004) 56(2):129–40. 10.1016/j.bandc.2004.09.016 15518930

[19] He Q Huang X Zhang S Turel O Ma L Bechara A Dynamic causal modeling of insular, striatal, and prefrontal cortex activities during a food-specific Go/NoGo task. Biol Psychiatry: Cogn Neurosci Neuroimaging (2019) 4(12):1080–9. 10.1016/j.bpsc.2018.12.005 PMC6609512 30691967

[20] Turel O He Q Wei L Bechara A The role of the insula in internet gaming disorder. Addict Biol (2020), e12894. 10.1111/adb.12894 32147952

[21] Miltner WH Braun CH Coles MG Event-related brain potentials following incorrect feedback in a time-estimation task: evidence for a “generic” neural system for error detection. J Cogn Neurosci (1997) 9(6):788–98. 10.1162/jocn.1997.9.6.788 23964600

[22] Franken IHA Strien JWV Kuijpers I Evidence for a deficit in the salience attribution to errors in smokers. Drug Alcohol Depend (2010) 106(2-3):181–5. 10.1016/j.drugalcdep.2009.08.014 19781864

[23] Franken I Van-Strien JE Van-De-Wetering B Error-processing deficits in patients with cocaine dependence. Biol Psychol (2007) 75(1):45–51. 10.1016/j.biopsycho.2006.11.003 17196732

[24] Sokhadze E Stewart C Hollifield M Tasman A Event-Related Potential Study of Executive Dysfunctions in a Speeded Reaction Task in Cocaine Addiction. J Neurother (2008) 12(4):185–204. 10.1080/10874200802502144 19830263 PMC2760844

[25] Shaoping S Dahua Y Limei B Yao M Chen Y Zhang X The changes of EEG signals during resting state in adolescents with smoking addiction. Chin J Behav Med Brain Sci (2017) 26(11):1021–4. 10.3760/cma.j.issn.16746554.2017.11.012

[26] Fagerstrom KO Schneider NG Measuring nicotine dependence: A review of the Fagerstrom Tolerance Questionnaire. J Behav Med (1989) 12(2):159–82. 10.1007/bf00846549 2668531

[27] Cox LS Tiffany ST Christen AG Evaluation of the brief questionnaire of smoking urges (QSU-brief) in laboratory and clinical settings. Nicotine Tobacco Res Off J Soc Res Nicotine Tobacco (2001) 3(1):7–16. 10.1080/14622200020032051 11260806

[28] Hughes JR Hatsukami D Signs and symptoms of tobacco withdrawal. Arch Gen Psychiatry (1986) 43(3):289–94. 10.1001/archpsyc.1986.01800030107013 3954551

[29] Syed TS Safety and Efficacy of the Nicotine Patch and Gum for the Treatment of Adolescent Tobacco Addiction. J Pediatr (2005) 115(4):e407–14. 10.1542/peds.2004-1894 15805342

[30] Eriksen BA Eriksen CW Effects of noise letters upon the identification of a target letter in a nonsearch task. Percept Psychophys (1974) 16(1):143–9. 10.3758/BF03203267

[31] Hajcak G McDonald N Simons RF Anxiety and error-related brain activity. Biol Psychol (2003) 64(1-2):0–90. 10.1016/s0301-0511(03)00103-0 14602356

[32] Schüller T Gruendler TOJ Huster R Baldermann JC Huys D Ullsperger M Altered Electrophysiological Correlates of Motor Inhibition and Performance Monitoring in Tourette’s Syndrome. Clin Neurophysiol (2018) 129(9):1866–72. 10.1016/j.clinph.2018.06.002 30005213

[33] Bernstein PS Scheffers MK Coles MG “Where did I go wrong?” A psychophysiological analysis of error detection. J Exp Psychol Hum Percept Perform (1995) 21(6):1312–22. 10.1037//0096-1523.21.6.1312 7490583

[34] Hajcak G What We’ve Learned From Mistakes Insights From Error-Related Brain Activity. Curr Dir psychol Sci (2012) 21(2):101–6. 10.1177/0963721412436809

[35] Ridderinkhof KR Ullsperger M Crone EA Nieuwenhuis S The role of the medial frontal cortex in cognitive control. Science (2004) 306(5695):443–7. 10.1126/science.1100301 15486290

[36] Debener S Ullsperger M Siegel M Fiehler K Von Cramon DY Engel AK Trial-by-trial coupling of concurrent electroencephalogram and functional magnetic resonance imaging identifies the dynamics of performance monitoring. J Neurosci (2005) 25(50):11730–7. 10.1523/JNEUROSCI.3286-05.2005 PMC6726024 16354931

[37] Vilà-Balló A Hdez-Lafuente P Rostan C Cunillera T Rodriguez-Fornells A Neurophysiological correlates of error monitoring and inhibitory processing in juvenile violent offenders. Biol Psychol (2014) 102(1):141–52. 10.1016/j.biopsycho.2014.07.021 25108171

[38] Padilla ML Colrain IM Sullivan EV Mayer BZ Turlington SR Hoffman LR Electrophysiological evidence of enhanced performance monitoring in recently abstinent alcoholic men. Psychopharmacology (2011) 213(1):81–91. 10.1007/s00213-010-2018-1 20941595 PMC3015191

[39] Ullsperger M Harsay HA Wessel JR Ridderinkhof KR Conscious perception of errors and its relation to the anterior insula. Brain Struct Funct (2010) 214(5-6):629–43. 10.1007/s00429-010-0261-1 PMC2886909 20512371

[40] Heishman SJ Kleykamp BA Singleton EG Meta-analysis of the acute effects of nicotine and smoking on human performance. Psychopharmacology (2010) 210(4):453–69. 10.1007/s00213-010-1848-1 PMC3151730 20414766

[41] Zijlmans J Bevaart F Van Duin L Luijks MJA Popma A Marhe R Error-related brain activity in relation to psychopathic traits in multi-problem young adults: An ERP study. Biol Psychol (2019) 144:46–53. 10.1016/j.biopsycho.2019.03.014 30928622

[42] Steele VR Maurer JM Bernat EM Calhoun VD Kiehl KA Error-related processing in adult males with elevated psychopathic traits. Pers Disord (2016) 7(1):80–90. 10.1037/per0000143 PMC4710563 26479259

[43] Overbye K Walhovd KB Paus T Fjell AM Huster RJ Tamnes CK Error processing in the adolescent brain: Age-related differences in electrophysiology, behavioral adaptation, and brain morphology. Dev Cogn Neurosci (2019) 38:100665. 10.1016/j.dcn.2019.100665 31176282 PMC6969341

[44] Leuthold H Sommer W ERP correlates of error processing in spatial S-R compatibility tasks. Clin Neurophysiol Off J Int Fed Clin Neurophysiol (1999) 110(2):342–57. 10.1016/s1388-2457(98)00058-3 10210624

[45] Falkenstein M Hoormann J Christ S Hohnsbein J ERP components on reaction errors and their functional significance: a tutorial. Biol Psychol (2000) 51(2-3):87–107. 10.1016/s0301-0511(99)00031-9 10686361

[46] Overbeek TJM Nieuwenhuis S Ridderinkhof KR Dissociable Components of Error Processing: On the Functional Significance of the Pe Vis-à-vis the ERN/Ne. J Psychophysiol (2005) 19(4):319–29. 10.1027/0269-8803.19.4.319

[47] Luijten M CSv M Franken IHA Diminished error processing in smokers during smoking cue exposure. Pharmacol Biochem Behav (2011) 97(3):514–20. 10.1016/j.pbb.2010.10.012 21047524

[48] Sucec J Herzog M Diest IV Bergh OVd Leupoldt Av The impact of dyspnea and threat of dyspnea on error processing. Psychophysiology (2018) 56(1):e13278. 10.1111/psyp.13278 30252140

[49] Parrott AC Garnham NJ Wesnes K Pincock C Cigarette smoking and abstinence: comparative effects upon cognitive task performance and mood state. Hum Psychopharmacol: Clin Exp (1996) 11(5):391–400. 10.1002/(SICI)1099-1077(199609)11:5<391::AID-HUP780>3.0.CO;2-Z

